# Correction: Gene expression profiling identifies FYN as an important molecule in tamoxifen resistance and a predictor of early recurrence in patients treated with endocrine therapy

**DOI:** 10.1038/s41388-019-1096-8

**Published:** 2019-11-11

**Authors:** D. Elias, H. Vever, A.-V. Lænkholm, M. F. Gjerstorff, C. W. Yde, A. E. Lykkesfeldt, H. J. Ditzel

**Affiliations:** 1 Department of Cancer and Inflammation Research, Institute of Molecular Medicine, University of Southern Denmark, Odense, Denmark; 2 Department of Pathology, Slagelse Hospital, Slagelse, Denmark; 3 Breast Cancer Group, Cell Death and Metabolism, Danish Cancer Society Research Center, Copenhagen, Denmark; 4 Department of Oncology, Odense University Hospital, Odense, Denmark

**Correction to: Oncogene**

10.1038/s41388-018-0495-6

Since the publication of this Erratum, the Authors noticed a further error in Fig. 1c. The same image was inserted twice next to each other (second row: PRKCA, image for RamR7 and TamR8) instead of the correct image for TamR8. The corrected Fig. 1c has been provided below.

**Figure 1:**
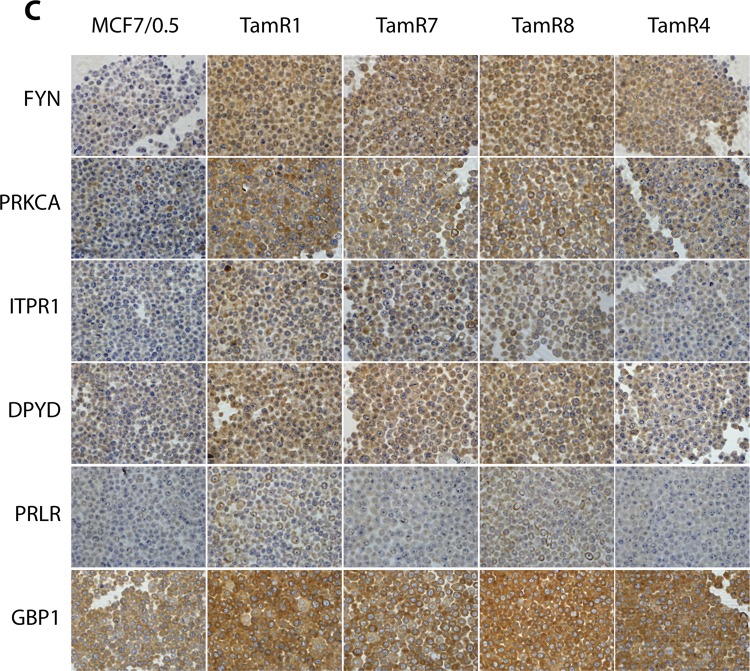
Fig. 1

